# The prevention and control of tuberculosis: an analysis based on a tuberculosis dynamic model derived from the cases of Americans

**DOI:** 10.1186/s12889-020-09260-w

**Published:** 2020-07-28

**Authors:** Yan Wu, Meng Huang, Ximei Wang, Yong Li, Lei Jiang, Yuan Yuan

**Affiliations:** 1 School of Information and Mathematics, Yangtze University, Jingzhou 434023, China, Nanhuan Road, Jingzhou, 434023 China; 2 Institute of Applied Mathematics, Yangtze University, Nanhuan Road, Jingzhou, 434023 China; 3 Department of Respiratory Medicine, Jingzhou Hospital of Traditional Chinese Medicine, Jiangjin East Road, Jingzhou, 434000 China; 4 Laboratory Department, Jingzhou Maternal and Child Health Hospital, Jingzhong Road, Jingzhou, 434000 China

**Keywords:** Tuberculosis (TB), Latin hypercube sampling (LHS), Partial rank correlation coefficients (PRCC), Basic reproduction number, Parameter estimation, Prevention and control measures

## Abstract

**Background:**

Tuberculosis (TB), a preventable and curable disease, is claimed as the second largest number of fatalities, and there are 9,025 cases reported in the United States in 2018. Many researchers have done a lot of research and achieved remarkable results, but TB is still a severe problem for human beings. The study is a further exploration of the prevention and control of tuberculosis.

**Methods:**

In the paper, we propose a new dynamic model to study the transmission dynamics of TB, and then use global differential evolution and local sequential quadratic programming (DESQP) optimization algorithm to estimate parameters of the model. Finally, we use Latin hypercube sampling (LHS) and partial rank correlation coefficients (PRCC) to analyze the influence of parameters on the basic reproduction number ($\mathcal R_{0}$) and the total infectious (including the diagnosed, undiagnosed and incomplete treatment infectious), respectively.

**Results:**

According to the research, the basic reproduction number is computed as 2.3597 from 1984 to 2018, which means TB is also an epidemic in the US. The diagnosed rate is 0.6082, which means the undiagnosed will be diagnosed after 1.6442 years. The diagnosed will recover after an average of 1.9912 years. Moreover, some diagnosed will end the treatment after 1.7550 years for some reason. From the study, it’s shown that 2.40% of the recovered will be reactivated, and 13.88% of the newborn will be vaccinated. However, the immune system will be lost after about 19.6078 years.

**Conclusion:**

Through the results of this study, we give some suggestions to help prevent and control the TB epidemic in the United States, such as prolonging the protection period of the vaccine by developing new and more effective vaccines to prevent TB; using the Chemoprophylaxis for incubation patients to prevent their conversion into active TB; raising people’s awareness of the prevention and control of TB and treatment after illness; isolating the infected to reduce the spread of TB. According to the latest report in the announcement that came at the first WHO Global Ministerial Conference on Ending tuberculosis in the Sustainable Development Era, we predict that it is challenging to control TB by 2030.

## Background

To date, tuberculosis (TB) is regarded as one of the world’s deadliest diseases caused by a single infectious agent, second only to acquired immunodeficiency syndrome (AIDS) caused by human immunodeficiency virus (HIV) [1, 2]. TB is caused by Mycobacterium tuberculosis (MTB), which can be expelled into the air when the infected people cough, talk, sneeze or sing [3]. In most situations, MTB generally affects the lungs of infected individuals. Tuberculosis is so highly contagious that the susceptible are likely to develop TB even when they inhaled tiny particles containing MTB. The MTB is very small and can stay in the air for a long time and keep moving. The immune system is an essential defense mechanism that limits the growth and spread of MTB. If the immune system cannot suppress their growth, they will most likely spread throughout the body [4]. In fact, not all people infected with TB will be sick at once. Some people’s incubation period may last from 1 year to several years. Nowadays, reliable TB tests do not exist [5], which causes many undiagnosed infectious and increases the difficulty in controlling TB.

It was reported in the Global Tuberculosis Report 2018 that there were 1.3 million deaths caused by TB [2]. People may be infected with TB in all countries and age groups, but overall, 90% were adults (aged ≥15 years), 9% were people living with HIV (72% in Africa). Most cases happened in following eight countries: India (27%), China (9%), Indonesia (8%), the Philippines (6%), Pakistan (5%), Bangladesh (4%), Nigeria (4%), South Africa (3%). WHO listed all of these countries and 22 other countries as the top 30 countries with the high burden of TB, which account for 87% of the world’s cases. On the contrary, the global cases of the WHO European Region (3%) and the WHO American Region (3%) account for only 6% [2].

Although the number of people infected with TB has been declining, there are about 10 to 20 thousands cases of TB every year in the last 20 years (see Table 1) [6], and the death rate is between 0.05 and 0.07 in America. Therefore, it is essential for researchers to explore the factors related to the infection, outbreak, and epidemic of TB so that we could take more measures to protect people from TB.

**Table 1:** Population, tuberculosis Cases, Case Rates per 100,000 Population, Deaths, and Death Rates per 100,000 Population, and Percent Change: The United States, 1984-2017. Where, $Rate^{i1}_{j}=\frac {Number^{i1}_{j}}{Number_{j}}$, $Number^{i2}_{j}=\frac {Number^{i1}_{j}-Number^{i1}_{j-1}}{Number^{i1}_{j-1}}$, $Rate^{i2}_{j}=\frac {Rate^{i1}_{j}-Rate^{i1}_{j-1}}{Rate^{i1}_{j-1}}=(\frac {Number^{i1}_{j}} {Number_{j}}-\frac {Number^{i1}_{j-1}}{Number_{j-1}})/\frac {Number^{i1}_{j-1}}{Number_{j-1}}$, (*i*=1,2) and *j* is the year. For example, 2016, 2.9=9253/3.2307×10^3^; −3.1=(9253−9547)/9547; −3.8=(9253/3230.7−9547/3207.4)/(9547/3207.4). The data of population from [7] and others from [6].

	Population	Tuberculosis Cases				Tuberculosis Deaths			
		Original		Change rate		Original		Change rate	
Year	Number (×10^9^)	*Number*^(11)^	*Rate*^(11)^	*Number*^(12)^	*Rate*^(12)^	*Number*^(21)^	*Rate*^(21)^	*Number*^(22)^	*Rate*^(22)^
1984	2.3583	22255	9.4	−6.7	−7.5	1729	0.7	−2.8	−3.6
1985	2.3792	22201	9.3	−0.2	−1.1	1752	0.7	1.3	0.4
1986	2.4013	22768	9.5	2.6	1.6	1782	0.7	1.7	0.8
1987	2.4229	22517	9.3	−1.1	−2.0	1755	0.7	−1.5	−2.4
1988	2.4450	22436	9.2	−0.4	−1.3	1921	0.8	9.5	8.5
1989	2.4682	23495	9.5	4.7	3.7	1970	0.8	2.6	1.6
1990	2.4962	25701	10.3	9.4	8.2	1810	0.7	−8.1	−9.2
1991	2.5298	26283	10.4	2.3	0.9	1713	0.7	−5.4	−6.6
1992	2.5651	26673	10.4	1.5	0.1	1705	0.7	−0.5	−1.8
1993	2.5992	25102	9.7	−5.9	−7.1	1631	0.6	−4.3	−5.6
1994	2.6313	24206	9.2	−3.6	−4.7	1478	0.6	−9.4	−10.5
1995	2.6628	22726	8.5	−6.1	−7.2	1336	0.5	−9.6	−10.7
1996	2.6939	21210	7.9	−6.7	−7.8	1202	0.4	−10.0	−11.1
1997	2.7266	19751	7.2	−6.9	−8.0	1166	0.4	−3.0	−4.2
1998	2.7585	18286	6.6	−7.4	−8.5	1112	0.4	−4.6	−5.7
1999	2.7904	17499	6.3	−4.3	−5.4	930	0.3	−16.4	−17.3
2000	2.8216	16308	5.8	−6.8	−7.8	776	0.3	−16.6	−17.5
2001	2.8497	15945	5.6	−2.2	−3.2	764	0.3	−1.5	−2.5
2002	2.8763	15055	5.2	−5.6	−6.5	784	0.3	2.6	1.7
2003	2.9011	14835	5.1	−1.5	−2.3	711	0.2	−9.3	−10.1
2004	2.9281	14499	5.0	−2.3	−3.2	657	0.2	−7.6	−8.4
2005	2.9552	14065	4.8	−3.0	−3.9	648	0.2	−1.4	−2.3
2006	2.9838	13727	4.6	−2.4	−3.3	652	0.2	0.6	−0.3
2007	3.0123	13280	4.4	−3.3	−4.2	554	0.2	−15.0	−15.8
2008	3.0409	12889	4.2	−2.9	−3.9	585	0.2	5.6	4.6
2009	3.0677	11514	3.8	−10.7	−11.4	529	0.2	−9.6	−10.4
2010	3.0932	11100	3.6	−3.6	−4.4	569	0.2	7.6	6.7
2011	3.1156	10504	3.4	−5.4	−6.1	539	0.2	−5.3	−6.0
2012	3.1383	9935	3.2	−5.4	−6.1	510	0.2	−5.4	−6.1
2013	3.1599	9561	3.0	−3.8	−4.4	555	0.2	8.8	8.1
2014	3.1830	9398	2.9	−1.7	−2.4	493	0.2	−11.2	−11.8
2015	3.2064	9547	3.0	1.6	0.8	470	0.1	−4.7	−5.4
2016	3.2294	9253	2.9	−3.1	−3.8	528	0.2	12.3	11.5
2017	3.2499	9105	2.8	−1.6	−2.3	515	0.2	−2.5	−3.1
2018	3.2669	9025	2.8	−0.7	−1.3	−	−	−	−

From 1945 to 1955, the widespread usage of antibiotics reduced TB mortality in the United States by 70%, but the United States still has a severe TB epidemic [4, 8]. Based on decades of technology and experience, most active and latent TB can be effectively treated, and latent TB can be treated with Isoniazid. However, treatment can be effective only if the course of cures lasts no less than six months [4]. Active TB can be eliminated with a complex treatment regimen, coupled with the treatment of multiple drugs (Isoniazid, Rifampicin, Pyrazinamide) for nine months [4, 8].

In the United States, people who contract TB and receive treatment fall into two categories [9, 10]. The first type of people are those who complete the treatment and eventually recover. The other type of the infected people do not complete treatment, due in part to lost data, patients’ adverse reactions to the drug, or their refusal of treatment, etc. Another potential reasons that causes incomplete treatment is the high cost of TB treatment, which means a heavy burden to the general public. When the treatment is incomplete, the drug-resistant strains will reproduce, which may severely increase the difficulty of treatment [4, 11, 12]. According to the latest treatment outcome data for new cases in 2016, 82% of the people can successfully recover from TB. Apart from the reduction from 2015 to 2016, there is another reduction from 86% in 2013 to 83% in 2015 [2].

Bacillus Calmette-Guerin (BCG) is a vaccine that has been used to prevent TB for a long time. The protection period of BCG vaccine usually varies from 10 to 20 years [13]. BCG precludes nearly 20 percent of children from getting infected, while the vaccine prevents approximately half of those already infected from getting worse [14]. The study of Fjallbrant et al. [15] suggested that the primary vaccination and revaccination of negative tuberculin skin test (TST) of young adults with BCG caused a significant increase in the T-helper 1 (Th1) response against mycobacterial antigens, suggesting a protective effect against TB. This gives support for the policy of primary vaccination as well as the revaccination under this setting and age group. In some areas where TB is prevalent, the primary BCG vaccination is essential, though should not be the primary measure for TB control. BCG is widely used in countries with a high burden of TB, but not those with a small burden. In the United States, BCG is often used by special people [16] but not widely used by the general public. As an alternative, Chemoprophylaxis [17, 18] is more affordable, easier to take, and can prevent latent infectious from turning into active TB. Thus, for some countries less inflicted by TB, Chemoprophylaxis is a better alternative to control TB [19].

On the dynamic model study, many researchers have devoted big efforts for the research of the epidemic law and transmission dynamics of TB. In 1962, Waaler et al. established the first dynamic model of TB based on a susceptible-infected-recovered (SIR) model [20]. From then on, many models that consider multiple influencing factors have been established, such as reinfection [21–23], vaccination [24–26], interactions with HIV [27, 28], reactivated [29, 30], Chemoprophylaxis [19] and so on [31–34]. Revelle et al. considered prophylaxis, cure and BCG vaccination to research the optimal strategy to fight against TB, which was then extensively used to study the epidemic model of transmission for infectious disease in 1967 [35]. Buonomo et al. studied the global behavior of a non-linear susceptible-infectious-removed (SIR)-like epidemic model with a non-bilinear feedback mechanism [36]. The SEI model proposed by Bowong et al. exhibits the traditional threshold behavior [34]. Whang et al. used an SEIR model with the time-dependent parameters to develop a dynamic model for TB transmission in South Korea [37]. A mathematical model was proposed to understand the spread of TB disease in the human population for both pulmonary and drug-resistant subjects by Mishra et al. [38]. Three control factors must be considered simultaneously to decrease the threat of TB by Gao et al.: a preventive measure in the form of vaccination and two treatment measures aiming at the susceptible and individuals infected TB in the active stage and latent stage [39].

In developing countries, the increase of TB cases by a high level of undiagnosed infectious population and incompletely treated population is one of the greatest challenges to control TB. These people are more likely to develop multi-drug resistance relative to the diagnosed infectious population [21, 40]. According to the actual situation of TB in the United States, we considered several factors: slow-fast process [41–43], vaccination [24–26], reinfection [21–23], reactivated [29, 30] and undiagnosed infection [21, 44]. Then we referenced the modeling thought from D.P. Moualeu et al. [21, 45] and Liu et al. [24], and finally established our model. The biggest differences between our model and their model are the vaccination and recovery to the susceptible population [21, 24, 45].

Our study aims to analyze the factors affecting TB based on the dynamics model, give several measures to control and prevent TB, and predict the epidemic trend in America. The structure of this paper is as follows. In Section 2, we introduce our TB model expressed by ordinary differential equations (ODE) and define parameters. Then we describe the model assumptions and modeling ideas in detail and give the disease-free equilibrium and basic reproduction number. In Section 3, the model is simulated by global differential evolution and local sequential quadratic programming (DESQP) [46, 47] optimization algorithm based on the US cases. We analyze the fitting effect by the root mean square percentage error (RMSPE) and the mean absolute percentage error (MAPE). In Section 4, we make the uncertainty and sensitivity analysis of the parameters for our model by Latin hypercube sampling (LHS) and partial rank correlation coefficients (PRCC). We also analyze the sensitivity of each parameter on the basic reproduction number and the total infectious, respectively. In Section 5 and 6, we analyze the results, make some suggestions to prevent TB, carry out simulation experiments, and then discuss the deficiency of our study. Finally, we summarize our research.

## Methods

In this section, we introduce our new mathematical model, briefly explain the structure of our model, and then analyze the basic reproduction number.

### 2.1 Model instruction

The total population is denoted by *N*(*t*), which is sub-divided into the following seven sub-populations:

*V*(*t*) vaccinated: healthy people vaccinated with TB,

*S*(*t*) susceptible: healthy people not exposed to TB,

*E*(*t*) exposed: exposed to TB but not infectious,

*I*(*t*) diagnosed infectious: infected with TB and diagnosed in hospital,

*J*(*t*) undiagnosed infectious: infected with TB but undiagnosed in hospital,

*L*(*t*) incompletely treated: have been diagnosed with active TB and begun their treatment in hospital or home, but quitted before the end,

*R*(*t*) recovered: recovered from TB after treatment.

**Fig. 1:**
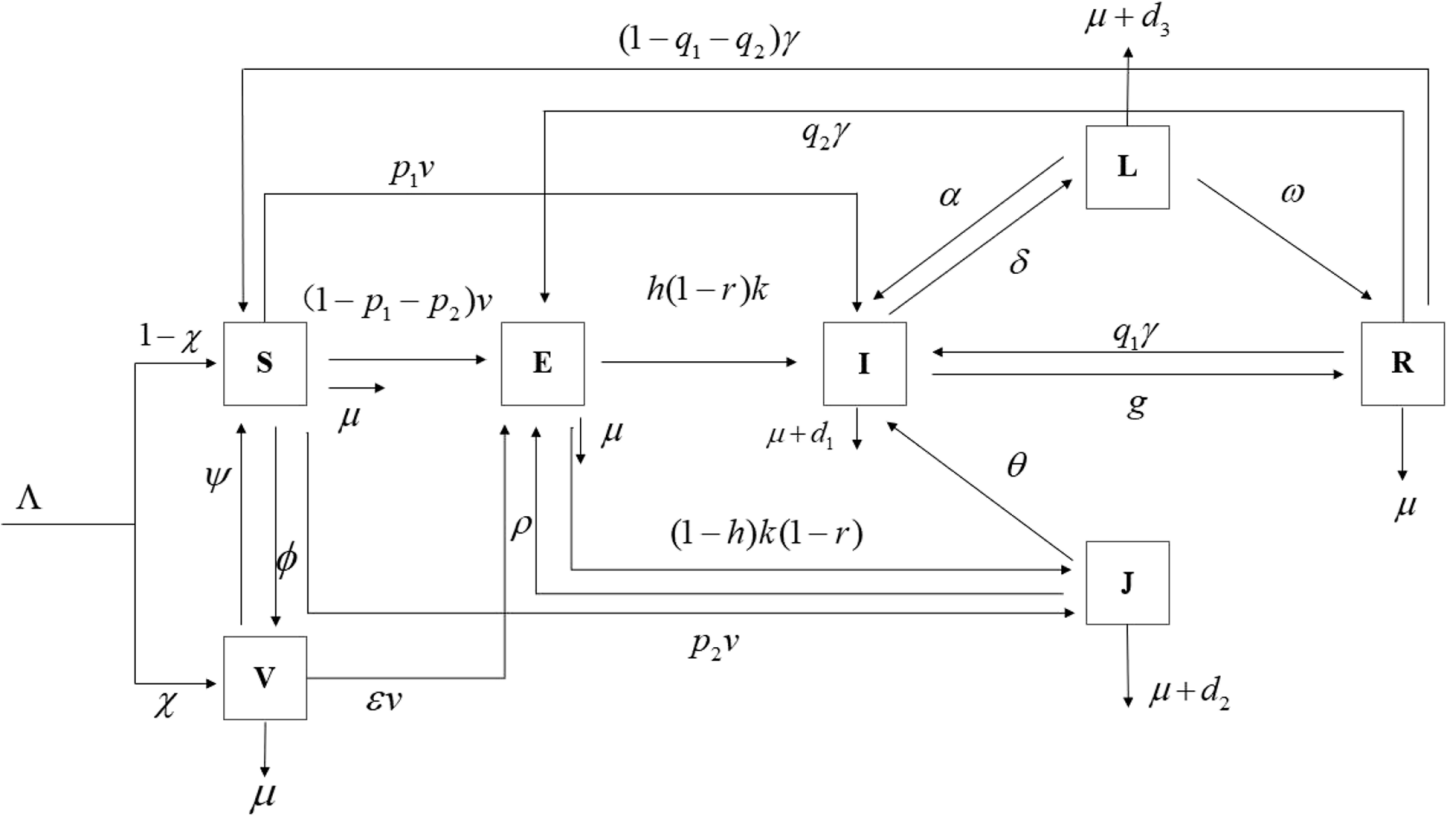
The flow diagram for the compartment model of the transmission dynamics system of TB

Considering slow-fast process [41–43], vaccination [24–26], reinfection [21–23], re-activated [29, 30] and undiagnosed infection [21, 44] and referencing the modeling thought of the D.P. Moualeu et al. [21, 45] and Liu et al. [24], we display our dynamic model with a flow diagram shown in Fig. 1 and introduce the model in detail as the following:

For the dynamic system, there will be a recruitment to the dynamic system with an average scale *Λ*. Considering the vaccination, a proportion *χ* will be vaccinated (i.e., primary vaccination) and become the vaccinated class, and the remainder (1−*χ*) will not be vaccinated and become the susceptible class.

In fact, BCG will be ineffective for some newborns, and those newborns will be infected by contacting with the infectious, we assume the fraction as *ε*. The vaccine protection period takes 10 to 20 years [13], during which there will be a rate *ψ* of losing the immune system and converging into the susceptible.

During the fast-slow process, the susceptible will be the exposed by contacting at a slow process (1−*p*) and turning into the active TB at a fast *p*. Because the test of TB is not sensitive, there will be a fraction *f* of diagnosed and (1−*f*) undiagnosed. Here, we set *p*_1_=*pf* and *p*_2_=*p*(1−*f*). We do not rule out that there will be a rate *ϕ*, at which the susceptible will be revaccinated and become the vaccinated.

For the exposed, a proportion *r* will use Chemoprophylaxis to prevent them from becoming active TB, and the remainder (1−*r*) will be the active TB at a rate *k*. For the active TB, there will be a fraction *h* of individuals who will be diagnosed and healed, and thus (1−*h*) undiagnosed.

In the diagnosed infectious, some will be recovered at a rate *g*. Some will be incompletely treated at a rate *δ*. For the undiagnosed, some will become diagnosed and go to the hospital at a rate *θ*; thanks to their immune system, some individuals will be the exposed at a rate *ρ*.

For the incomplete treatment, some will detect their illness and go to the hospital at a rate *α*, while others will become recovered with the aid from self-immunity at a rate *ω*.

Considering the re-infected and reactivated, the recovered will lose the immune system at a rate *γ*. A fraction *q*_1_ of recovered people will be reactivated and become the diagnosed infectious; a fraction *q*_2_ of the recovered will be re-infected but not be the active; and the remaining recovered individuals with a fraction 1−*q*_1_−*q*_2_ will become the susceptible.

Lastly, the death of a certain number of people in each part of the system is resulted from natural causes. In our article, the main feature of our model is to consider the transmission route and mechanism of TB in the context of reality in a comprehensive way, which is of a great practical significance. Therefore, the conclusion will be closer to reality.

### 2.2 Model introducing

We give the definition and range of the parameters in Table 2, and proposed a mathematical model to state the transmission dynamics and epidemic of TB which is represented by the following system of ordinary differential equations:

1 $$\begin{array}{@{}rcl@{}} {\begin{aligned} \left\{ \begin{array} {l} \frac{dV}{dt} = \Lambda \chi + \phi S - (\psi + \mu)V - \varepsilon vV,\\ \frac{dS}{dt} = \Lambda (1 - \chi) + \psi V + (1 - {q_{1}} - {q_{2}})\gamma R - \phi S - vS - \mu S,\\ \frac{dE}{dt} = \varepsilon vV + (1 - {p_{1}} - {p_{2}})vS + {q_{2}}\gamma R + \rho J - (1 - r)kE - \mu E,\\ \frac{dI}{dt} = {p_{1}}vS + h(1 - r)kE + \theta J + {q_{1}}\gamma R + \alpha L - (\delta + g)I - (\mu + {d_{1}})I,\\ \frac{dJ}{dt} = (1 - h)k(1 - r)E + {p_{2}}vS - (\theta + \rho)J - (\mu + {d_{2}})J,\\ \frac{dL}{dt} = \delta I - (\alpha + \omega)L - (\mu + {d_{3}})L,\\ \frac{dR}{dt} = gI + \omega L - (\gamma + \mu)R,\\ \end{array}\right. \end{aligned}}  \end{array} $$

**Table 2:** The definition and value range of parameters for the model (1)

Parameter	Definition
*μ*	Natural mortality of human
*Λ*	New individuals coming into the system
*α* in [0.1,0.4]	The rate for incompletely treated going to diagnosed infectious
*β*_1_ in [2,6]	The transmission rate of diagnosed infectious
*β*_2_ in [0.1,0.4]	The transmission rate of undiagnosed infectious
*β*_3_ in [4,6]	The transmission rate of incompletely treated
*χ* in [0.08,0.3]	The natural vaccination ratio of the newborn babies
*d*_1_ in [0.05,0.1]	TB-related mortality of diagnosed infectious
*d*_2_ in [0.5,0.8]	TB-related mortality of undiagnosed infectious
*d*_3_ in [0.09,0.3]	TB-related mortality of incompletely treated
*δ* in [0.5,0.8]	Rate for diagnosed infectious coming into the incompletely treated
*ε* in [0,0.3]	Indicate the reduction in risk of infection due to vaccination
*g* in [0.4,0.8]	Recovery rate of the diagnosed infectious
*γ* in [0.125,0.6]	Reactivated rate of recovered individuals
*h* in [0.2,0.6]	Detection ratio of active TB
*k* in [0.033,0.333]	Rate of progression to infectious
*p*_1_ in [0.02,0.08]	The proportion of susceptible individuals who become diagnosed infectious
*p*_2_ in [0,0.5]	The proportion of susceptible individuals who become undiagnosed infectious
*ψ* in [0.05,0.2]	Loss of vaccination rate
*ϕ* in [0.05,0.1]	Vaccine coverage rate
*q*_1_ in [0,0.4]	The ratio of recovered relapsing into the infectious
*q*_2_ in [0.15,0.6]	The ratio of recovered reinfected into the exposure
*r* in [0.7,0.9]	Chemoprophylaxis of the exposed
*ρ* in [0.15,0.25]	Rate of progression from undiagnosed infectious to exposed
*θ* in [0.6,0.9]	Rate of progression from undiagnosed infectious to diagnosed infectious
*ω* in [0.15,0.25]	Recovery rate of the incompletely treated

where

2 $$ N(t) = V(t) + S(t) + E(t) + I(t) + J(t) + L(t) + R(t).  $$

The susceptible and the vaccinated are infected with TB through contacting individuals with active TB at a rate *v*(*I*,*J*,*L*) given by:

3 $$ v = \frac{{{\beta_{1}}I}}{N} + \frac{{{\beta_{2}}J}}{N} + \frac{{{\beta_{3}}L}}{N},  $$

where *β*_*i*_, *i*=1,2,3 are the rates that the diagnosed, undiagnosed infectious, and incompletely treated people sufficiently and effectively transmit TB to the susceptible or the vaccinated [1].

### 2.3 Basic reproduction number

The basic reproduction number ${\mathcal R_{0}}$ represents the number of infected during the initial patient’s infectious (not sick) period [48]. Our model is a biological system model, so it must meet the biological conditions. Therefore, we only study the dynamic state of the solution of system (1) in the following feasible region:

4 $$ {\begin{aligned} \Omega = \left\{ (V,S,E,I,J,L,R) \in \mathbb{R}_{+}^{7}:V + S + E + I + J + L + R \leq \frac{\Lambda}{\mu}\right\}, \end{aligned}}  $$

which can be confirmed as positively invariant. Therefore, we restrict our attention to the dynamics of the model (1) in *Ω*.

For the threshold system, when ${\mathcal R_{0}}<1$, the model will stabilize to the disease-free equilibrium, and the disease will be controlled and eventually become extinct. When ${\mathcal R_{0}}>1$, the model will stabilize to the endemic equilibrium, and the disease will develop into an endemic disease. For other complex systems, this conclusion may not be valid, for example, backward bifurcation, multistable system and other complex dynamic behaviors may occur. Therefore, the smaller ${\mathcal R_{0}}$ is, the easier to control TB [49, 50]. Here, we use the next-generation matrix approach to calculate the basic reproduction number ${\mathcal {R}_{0}}$, which was proposed by Van den Driessche, etc [49]. The detail calculation is given in the Appendix.

## Simulation

Based on the reported data from 1984 to 2018 by WHO [6] and the model (1), a global differential evolution and local sequential quadratic programming (DESQP) optimization algorithm was conducted to estimate the undetermined parameters [47, 51]. DESQP, which combines differential evolution (DE) [46] and local sequential quadratic programming (SQP) [52, 53], is a method used to search for the optimal solution of DE. In the method, DE is used as a base level search and SQP is used as a local search. DE is first applied to the short term of the problem to find the best solution. This optimal solution is given to SQP as an initial condition to fine tune the solution to reach the global optimum or near global optimum.

We get the estimated value, standard deviation, confidence interval, P-value and t-statistic of the parameters, as listed in Table 3. Based on the estimated results, the basic reproduction number ${\mathcal R_{0}}$ can be calculated ${\mathcal R_{0}}=2.3597$. Aandahl et al.[54] in 2014 specified an informative in [55] and improved the convergence performance of the Markov chain Monte Carlo (MCMC) sampler in [56], then estimated the reproduction number as: 2.1 (95% CI: 1.54-2.66) for the approximate method in [55] and 2.05 (95% CI: 1.55-2.63) for the exact method in [56].

**Table 3:** The t-statistic, P-value, CI Bound, Standard deviation, the estimated value of parameters, and the initial condition of each compartment of the model (1)

Parameter	Value	Standard	CI Low	CI High	P-Value	t-statistic
initial value		deviation	Bound	Bound		
*α*	0.1002	0.0340	-0.0081	0.2085	0.0603	2.9434
*β*_1_	4.4381	0.2390	3.6774	5.1987	0.0003	18.5687
*β*_2_	0.2994	0.0312	0.2002	0.3985	0.0024	9.6079
*β*_3_	5.8662	0.1946	5.2469	6.4855	0.0001	30.1466
*χ*	0.1388	0.0425	0.0036	0.2740	0.0469	3.2664
*d*_1_	0.0506	0.0240	-0.0256	0.1269	0.1251	2.1126
*d*_2_	0.7257	0.1033	0.3968	1.0546	0.0059	7.0222
*d*_3_	0.0947	0.0572	-0.0875	0.2769	0.1966	1.6543
*δ*	0.5698	0.1622	0.0535	1.0861	0.0391	3.5121
*ε*	0.5539	0.1130	0.1944	0.9134	0.0162	4.9029
*g*	0.5022	0.1049	0.1682	0.8362	0.0173	4.7856
*γ*	0.1444	0.0388	0.0210	0.2679	0.0337	3.7225
*h*	0.3998	0.0687	0.1811	0.6184	0.0101	5.8184
*k*	0.0421	0.0154	-0.007	0.0913	0.0721	2.7281
*Λ*	3.3510×10^3^	6.6920	3.3300×10^3^	3.3723×10^3^	1.7562×10^−8^	5.0076×10^2^
*p*_1_	0.025	0.0125	-0.0146	0.0647	0.1384	2.0072
*p*_2_	0.3414	0.0538	0.1702	0.5125	0.0079	6.3472
*ϕ*	0.0500	0.0255	-0.0311	0.1311	0.1444	1.9630
*ψ*	0.0510	0.0123	0.0120	0.0901	0.0252	4.1617
*q*_1_	0.0240	0.0077	6.7543 ×10^−4^	0.0486	0.0535	3.0952
*q*_2_	0.5425	0.0390	0.4183	0.6667	0.0008	13.9023
*r*	0.9219	0.0259	0.8394	1.0045	0.0000	35.5518
*ρ*	0.1993	0.0769	-0.0456	0.4442	0.0811	2.5903
*θ*	0.6082	0.1642	0.0856	1.1308	0.0342	3.7037
*ω*	0.1986	0.0233	0.1245	0.2726	0.0034	8.5350
*V*(0)	9.2311×10^5^	172.1804	9.2256×10^5^	9.2366×10^5^	1.4311×10^−11^	5.3613×10^3^
*S*(0)	4.6139×10^6^	101.8849	4.6136×10^6^	4.6142×10^6^	2.3746×10^−14^	4.5286×10^4^
*E*(0)	1.1199×10^6^	164.3129	1.1194×10^6^	1.1204×10^6^	6.9652×10^−12^	6.8157×10^3^
*I*(0)	2.2255×10^4^	−	−	−	−	−
*J*(0)	4.8511×10^4^	21.2908	4.8443×10^4^	4.8579×10^4^	1.8643×10^−10^	2.2785×10^3^
*L*(0)	5.7244×10^2^	2.5804	5.6423×10^2^	5.8065×10^2^	2.0199×10^−7^	2.2184×10^2^
*R*(0)	6.9875×10^2^	1.9114	6.9267×10^2^	7.0483×10^2^	4.5139×10^−8^	3.6557×10^2^

**Fig. 2:**
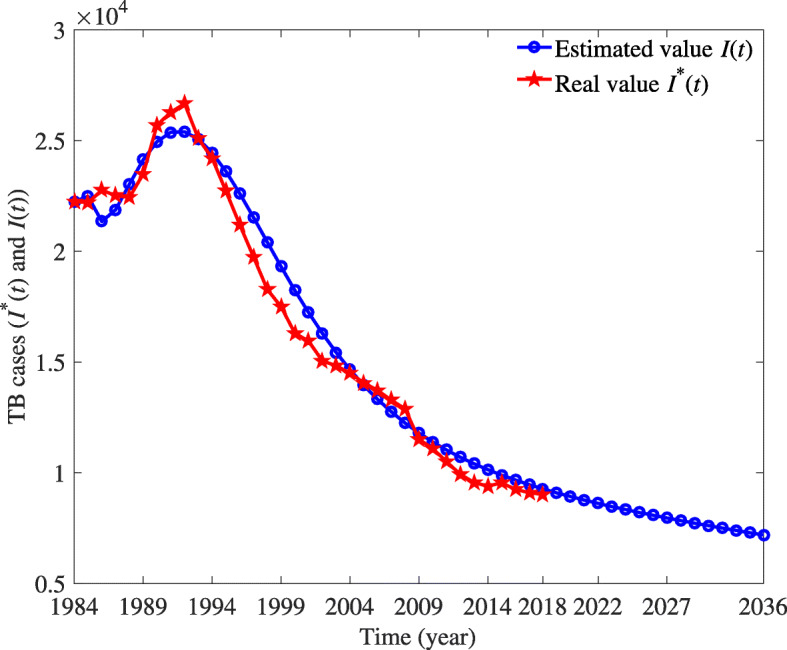
The comparison of real data and fitted data and the projection for the future status of TB

The real data and the model results are shown in the following Fig. 2. We evaluate the fitting effect of our established model through the root mean square percentage error (RMSPE) and the mean absolute percentage error (MAPE) which are significant evaluation indicators. The RMSPE and the MAPE are defined as:

5 $$ \text{MAPE} = \left({\frac{1}{n}\sum\limits_{t = 1}^{n} {\left| {\frac{{I{{(t)}^{*}} - I(t)}}{{I{{(t)}^{*}}}}} \right|}} \right) \times 100\%,  $$

6 $$ \text{RMSPE} = \sqrt {\frac{{\sum\limits_{t = 1}^{n} {{{\left[ {(I{{(t)}^{*}} - I(t))/I{{(t)}^{*}}} \right]}^{2}}} }}{{n - 1}}} \times 100\%,  $$

where *I*(*t*)^∗^ is the real value at time *t*, *I*(*t*) is its fitting value, and *n* is the number of data used for prediction. The criteria for MAPE and RMSPE are shown in Table 4 [57, 58]. We use model (1) to simulate the number of the infected, where MAPE=4.7245% and RMSPE=5.7676%, which means the fitting effect is very desirable and our system has strong prediction ability and high prediction accuracy.

**Table 4:** The criteria for MAPE and RMSPE

MAPE and RMSPE	Forecasting power
<10%	Highly accurate forecasting
10-20%	Good forecasting
20-50%	Reasonable forecasting
>50%	Inaccurate forecasting

## Sensitivity analysis

In model (1), the precise estimation of parameter values is one of the greatest challenges. Direct measurements of specific biological parameters are rare, and many parameters are estimated within a broad range of values identified by fitting the model with limited experimental data [59]. Therefore, the estimation of the parameters is always associated with uncertainty analysis (UA) and sensitivity analysis (SA). It is vital to study the influence of the uncertainty of these parameters on the model, as this will not only help us successfully use the mathematical and computational models of biological systems as prediction tools, but also comprehend the functions of biological systems.

Some factors are which we cannot control in the model (1). Generally, researchers would choose the factors that we can control to analyze the sensitivity so that we can find effective measures to eliminate TB. Since we cannot control the death rate *d*_1_, *d*_2_, and *d*_3_ in our analysis, we only did sensitivity analysis for 22 parameters that we can control in an attempt to eliminate TB. To find how the parameters impact on the outcome, a general and better way is to do the sensitivity analysis for each parameter. The ideal method is to use Latin Hypercube Sampling (LHS) and Partial Rank Correlation Coefficient (PRCC) to study the dependence of model parameters on the basic reproduction number and total infectious [60, 61].

### 4.1 Latin hypercube sampling (LHS)

Generally, the input factors of the most mathematical and computational model consist of initial conditions and parameters, which are independent and dependent model variables. Thanks to natural variation, lack of current techniques, measurement error, etc., the parameters are not always known with adequate certainty [61]. The purpose of uncertainty analysis (UA) is to solve these problems. UA can quantify the degree of confidence in the experimental data and the estimated values of the parameters [61].

In the article, introduced by Mckay et al., the most popular and efficient Latin hypercube sampling-LHS that belongs to Monte Carlo (MC) class of sampling methods was used to perform UA [61]. MC method, a common algorithm to solve various computational problems, can evaluate multiple models, the results of which can not only be used to perform SA, but also to determine the uncertainty of model inputs. LHS can unbiasedly estimate the average output of the model, and fewer samples are required to achieve the same accuracy as simple random sampling [61].

**Fig. 3:**
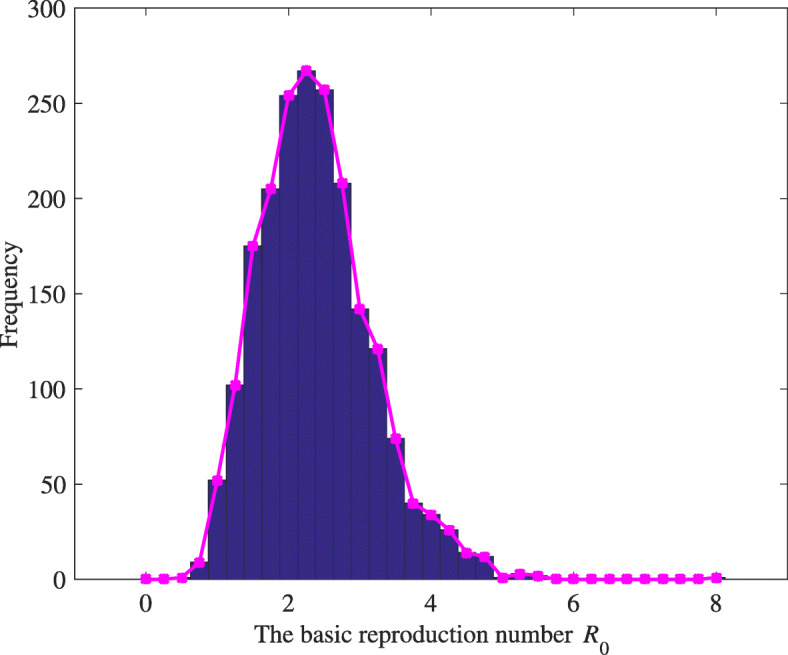
The distribution of the basic reproduction number ${\mathcal {R}_{0}}$

The remaining 22 parameters have been chosen to do uncertainty analysis. We assume each parameter to be a random variable with normal distribution to analyze the uncertainty in the value of these parameters. Normal distribution for all parameters with the mean (i.e., estimated value) and variance value (i.e., square of standard deviation) are given in Table 3. Latin hypercube sampling has been used to sample for these parameters considered for the sensitivity analysis. Here, we set the sample size N=2000. Using Latin hypercube sampling method and probability density function for each parameter is stratified into 2000 equiprobable (1/2000) serial intervals. Then a single value is randomly chosen from each interval. This produces 2000 sets of values for each parameter, and we can compute 2000 sets of values for $\mathcal R_{0}$ from 2000 sets of different parameter values and get the distribution hist of $\mathcal R_{0}$, as is shown in Fig. 3.

### 4.2 Partial rank correlation coefficient (PRCC)

Sensitivity analysis (SA) is a quantitative way to analyze the effects of the parameter uncertainty on the model’s outputs. Based on the parameters, we are able to raise presumptions about the biological system that actuates the system behavior, which can be measured by conducting experiments [62]. Local SA techniques, one class of SA, investigate on the effects of small variations in individual parameters around some nominal point and have been applied to a number of signal transduction and metabolic pathway models [63, 64]. Since the most influential parameters are determined, the predictability of the model can be significantly enhanced.

**Fig. 4:**
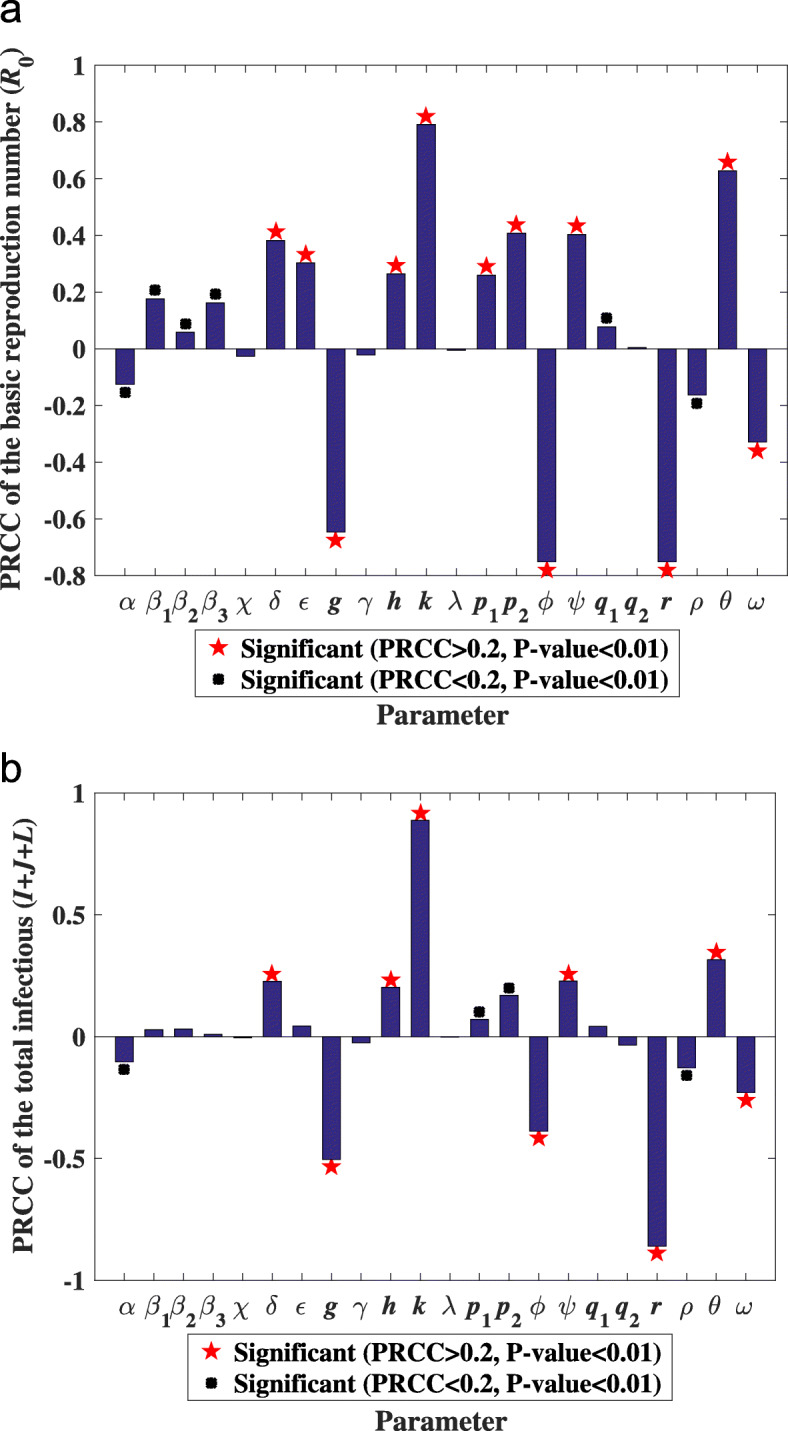
**(a)** show the PRCC of parameters with $\mathcal R_{0}$; **(b)** show the PRCC of parameters with the total infected. Here,we assume that when P-value <0.01, the parameters have significant effect $\mathcal R_{0}$ and the total infectious. To better control TB, we emphasize on analyzing the parameters whose PRCC >0.2

In this section, we compute PRCC to analyze the sensitivity of the parameters to the ${\mathcal {R}_{0}}$ and the total infectious so as to identify the parameters that have great effect on the variability in the outcome and how those parameters affect both ${\mathcal {R}_{0}}$ and the total infectious. Here we compute the PRCC of $\mathcal R_{0}$ and the total infectious based on the LHS matrix, the result of which can be seen from Fig. 4, Table 5. In our experiment, we assume that the parameters have a significant effect when P-value <0.01.

**Table 5:** The value of PRCC between each parameter and ${\mathcal {R}_{0}}$ and the total infectious

Parameters	${\mathcal {R}_{0}}$		Total infectious	
	PRCC	P-value	PRCC	P-value
*α*	-0.1025	4.8981 ×10^−6^	-0.1248	2.5216 ×10^−8^
*β*_1_	0.0281	2.1229 ×10^−1^	0.1756	2.5743 ×10^−15^
*β*_2_	0.0306	1.7314 ×10^−1^	0.0580	9.9135 ×10^−3^
*β*_3_	0.0096	6.7013 ×10^−1^	0.1614	5.1136 ×10^−13^
*χ*	-0.0050	8.2461 ×10^−1^	-0.0259	2.5024 ×10^−1^
*δ*	0.2269	1.5761 ×10^−24^	0.3822	7.6609 ×10^−70^
*ε*	0.0437	5.2022 ×10^−2^	0.3028	3.0568 ×10^−43^
*g*	-0.5037	7.7684 ×10^−128^	-0.6457	6.9717 ×10^−234^
*γ*	-0.0247	2.7261 ×10^−1^	-0.0220	3.2766 ×10^−1^
*h*	0.2023	1.0012 ×10^−19^	0.2636	8.0884 ×10^−33^
*k*	0.8883	0.0000	0.7931	0.0000
*Λ*	-0.0015	9.4530 ×10^−1^	-0.0051	8.2052 ×10^−1^
*p*_1_	0.0713	1.4980 ×10^−3^	0.2591	9.9110 ×10^−32^
*p*_2_	0.1699	2.7490 ×10^−14^	0.4083	2.3152 ×10^−80^
*ϕ*	-0.3874	6.9735 ×10^−72^	-0.7513	0.0000
*ψ*	0.2276	1.1225 ×10^−24^	0.4032	3.1138 ×10^−78^
*q*_1_	0.0423	5.9895 ×10^−2^	0.0776	5.4967 ×10^−4^
*q*_2_	-0.0341	1.2968 ×10^−1^	0.0046	8.3880 ×10^−1^
*r*	-0.8593	0.0000	-0.7511	0.0000
*ρ*	-0.1269	1.4744 ×10^−8^	-0.1633	2.6748 ×10^−13^
*θ*	0.3151	7.2127 ×10^−47^	0.6273	5.8105 ×10^−217^
*ω*	-0.2294	4.7563 ×10^−25^	-0.3289	3.9428 ×10^−51^

From Fig. 4, we can easily see the different parameters have different extent of the effect on both ${\mathcal R_{0}}$ and the total infectious, so it is complex to take proper measures to control TB. To better control TB, we emphasize on analyzing the parameters whose PRCC >0.2. Apart from that, we assume that these parameters have a high degree and a significant effect on ${\mathcal R_{0}}$ and the total infectious. Expect for the uncontrollable factor (which we cannot take relative measure to control TB) from Fig. 4, we can easily see that *r*, *ω*, *g* and *ϕ* have significantly positive affect on both the ${\mathcal R_{0}}$ and the total infectious, while *ε*, *k*, *ψ*, *δ* and *θ* have significantly negative effect on both the ${\mathcal R_{0}}$ and the total infectious.

## Results

In this section, we present the results of the simulation for the model. In general, parameter estimation is an iterative process, in which we use the current parameter values as the initial values of the next iteration [1]. All the parameter values of the first iterative process are set to be their initial guess values, which are estimated with the lowest sub-condition. Then parameters estimation is carried out with a limited list of previously non-identifiable parameters. Finally, we repeat the estimation process and check all the estimated parameters to see whether the new values of the previously unrecognized parameters affect the values of the identifiable parameters. We use the data of TB cases (i.e., diagnosed infectious) in America from 1984 to 2018 (see Table 1) published by the Centers for Disease Control and Prevention (CDC) to estimate the parameters of the model (1).

In our model, some parameters have been estimated by WHO, some evaluated by the TB researchers, and the others remain uncertain. We specify some parameter values as listed below.

(1) The natural mortality *μ*: It is assumed to be equal to the inverse of the life expectancy at birth and 1/*μ*=79.30 is the average human lifespan. Accordingly *μ*=0.0126 [65].

(2) Progress rate of the exposed to infectious individuals (including diagnosed and undiagnosed infectious) *k*: Based on the parameter estimation *k*=0.0421, the incubation period of TB is 1/*k*=23.7529 years. The latent TB infection lasts from 1 year to several years in general [1, 19, 66].

(3) Recovery rate of the diagnosed infectious *g*: In our simulation, *g*=0.5022, and the course of recovery for the diagnosed infection is estimated 1/*g*=1.9912 years. By 2010, the course of treatment of the first-time TB patient is normally treated in 6 months; and the course of treatment of the reinfected tuberculosis patient is usually 18-24 months [67, 68].

(4) Diagnosis rate *θ*: *θ*=0.6082 per year, which means the undiagnosed individual will be diagnosed with active TB after 1/*θ*=1.6442 years. Generally, some people with active TB are difficult to be diagnosed.

(5) Progression rate at which diagnosed infectious people become incompletely treated *δ*: It has been estimated as *δ*=0.5698 per years, which means the diagnosed people may give up treatment after 1/*δ*=1.7550 years. Generally, the average convalescence period of TB is around 1 year, which means after treated for 1 year, people will consider themselves to be recovered, but this is not the case [49].

(6) Reactivated ratio *q*_1_ and reinfected ratio *q*_2_: It has been estimated as *q*_1_=2.40*%*, *q*_2_=54.25*%*. This shows that relapse for most people is a slow progress, but re-infected is fast. (1−*q*_1_−*q*_2_)=43.35*%*, which means the people will lose the immune system and become the susceptible.

(7) Progress rate at which incompletely treated people become diagnosed infectious *α*: It has been estimated as *α*=0.1002, which means that the patients that are incompletely treated may be retreated after 1/*α*=9.9800 years.

(8) The natural vaccination rate of the newborn babies *χ*: It has been estimated as *χ*=13.88*%*. In America, only a few people get BCG vaccination [16–18]. The United States and other western countries with low TB burden do not necessarily require people to be vaccinated against BCG for newborns. Some Americans will be vaccinated per doctor’s advice [16–18].

(9) Progress rate *ψ*: the rate at which the vaccinated become the susceptible. It has been estimated as *ψ*=0.0510 which means the vaccination may be invalid after 1/*ψ*=19.6078 years. BCG vaccine duration varies widely, ranging from 10 to 20 years [13].

(10) Progress rate *γ*: the rate at which the recovered individuals lose the immune system. It is estimated as *γ*=0.1444 per year which means the recovered individuals may lose the immune system after 1/*γ*=6.9252 years.

(11) Progression rate at which the undiagnosed become the exposed *ρ*. It has been estimated as *ρ*=0.1993 which means it takes 1/*ρ*=5.0176 years or so for the undiagnosed to become non-infectious individuals (exposed).

(12) Detection rate of active TB *h*: It is estimated as *h*=39.98*%*. This shows that 60.02% of tuberculosis patients will not be diagnosed or will not be diagnosed for a short time.

(13) Vaccination coverage *ϕ*: It has been estimated as *ϕ*=0.0500, which means after an average of 1/*ϕ*=20.0000 years, people will lose antibodies to TB and be vaccinated again. Generally, BCG is not widely used in America, and the adults also will not choose to be vaccinated if it’s unnecessary [16, 17].

(14) Chemoprophylaxis rate *r*: It has been estimated as *r*=0.9219. Dye et al. have estimated *r*=0.7 [19, 69, 70].

(15) Recovery rate of the incompletely treated *ω*: It has been estimated as *ω*=0.1986, which means some incompletely treated individuals will naturally recover after 1/*ω*=5.0352 years. Bacaër et al. have estimated that the natural recovery from HIV-negative TB and HIV-positive TB takes 0.1390 and 0.2400 per year, respectively. HIV-negative TB and HIV-positive TB will be recovered after 7.1900 years and 4.1700 years without treatment [71].

(16) The rate of the susceptible become the diagnosed and undiagnosed infectious *p*_1_,*p*_2_: It is estimated that *p*_1_=2.5*%* and *p*_2_=34.14*%*, which means 2.5*%*+34.14*%*=36.54*%* of the people will be sick at once after infected by TB, while 2.5% have severe symptoms and 34.14% have mild symptoms and do not get a diagnosis. The remaining (1−*p*_1_−*p*_2_)=63.46*%* of people are those who proceed to a slow progression of TB infection become the exposed.

## Discussion

**Fig. 5:**
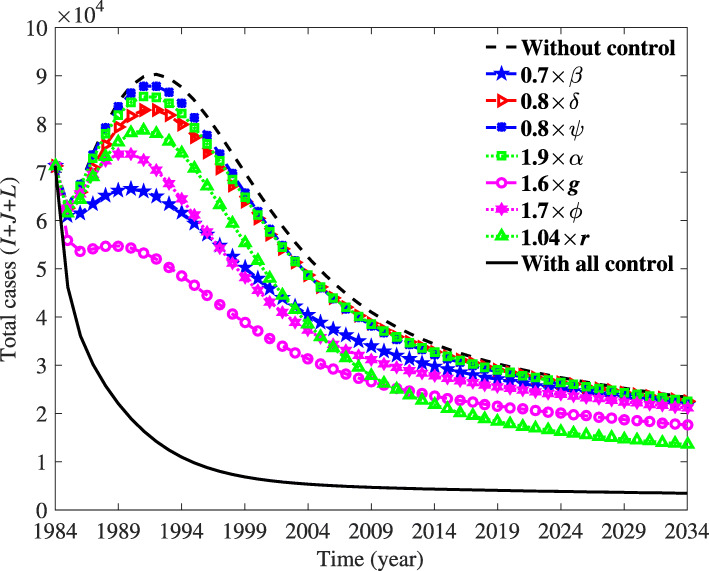
Simulation of the total infectious with parameters 1.03×*r*=0.9496, 1.5×*α*=0.1503, 1.5×*g*=0.7533, 1.5×*ϕ*=0.0750, 0.9×*δ*=0.5128, 0.99×*ψ*=0.0505, 0.7×*β*_1_=3.1067, 0.7×*β*_2_=0.2096 and 0.7×*β*_3_=4.1063, when one parameter takes a specific value, others take the value of the first column in Table 3. Differently, we synthesize the effects of three contact rates *β*_*i*_,*i*=1,2,3 into one contact rate *β* effect. ‘With all control’ means that we let all parameters specific values simultaneously. ‘Without control’ is the situation which we take no measures. We can find *ψ* has a mild effect on the total infected, with its line overlapping with ‘without control’ approximately

In the section, we discuss the sensitivity of parameters to the ${\mathcal R_{0}}$ and the total infectious. Furthermore, we suggest several measures to prevent TB. TB is a prevalent infectious disease in the world and the infectious are spread worldwide. It is vital to seize the leading causes and find the best measures to prevent and control the disease. In the article, we construct a TB model to study the transmission dynamic and provide some measures to control and prevent TB in the US. To find more ways to prevent TB, we analyze many factors that may have affect on ${\mathcal R_{0}}$ and the total infectious. Besides, we complete the sensitivity analysis of the parameters with ${\mathcal R_{0}}$ and the total infectious (see Fig. 4, Table 5). When we take measures to control TB, the result is shown in Fig. 5. In general, we find we can control the factors with *α*, *g*, *ψ*, *r*, *β*_*i*_,*i*=1,2,3, *ϕ* and *δ*. From the result in Fig. 5, we can find that *r* has the greatest effect on the total infected, then *g* has the second greatest effect, and others have a similar effect.

Strategy 1: We can find *r* is strongly negatively correlated with ${\mathcal R_{0}}$, which means it is wise to use Chemoprophylaxis to control TB. With our control, the TB can be significantly controlled from Fig. 5. In order to prevent the TB outbreak, we can encourage people to have Chemoprophylaxis by the media, research on new and more efficient chemoprophylaxis to improve the effect, reduce the harm to the human body, and improve the therapeutic effect [72].

Strategy 2: The parameter *g* has a negative effect on the ${\mathcal R_{0}}$, and with our control, it evidently reduced the total infectious. In doing so, we should take more money and energy to research on new medicine and therapy to reduce the period of treatment [73]. Nowadays, despite improvement in the treatment of TB, it remains the second leading cause of death in the world. Therefore, we still have a long way to eradicate TB.

Strategy 3: From Fig. 4, we can see *β*_*i*_,*i*=1,2,3 have a positive effect on ${\mathcal R_{0}}$. With our control, we can greatly reduce the total infectious by reducing the transmission rate. Although we have placed emphasis on treating TB patients in isolation the majority were infected TB through person-to-person contact each year [74]. We must strictly monitor, take protective measures to avoid TB, and examine outsiders to prevent contact with TB patients [75]. If we decrease half of *β*_*i*_,*i*=1,2,3, we can find the total infectious will drop (see Fig. 5).

Strategy 4: It is also clearly shown that *ψ* is positively correlated with ${\mathcal R_{0}}$ and the total infectious, which means the longer the vaccine lasts, the easier it is to control TB. We make 0.8×*ψ*, and find the total number of the infectious being reduced greatly, which means we can lower it to prevent TB. In addition to that, we can delay the duration of the BCG by researching on new and better vaccination to prevent infecting TB. Currently, one of the primary health interventions that can be used to prevent TB is to vaccinate children.

Strategy 5: The parameter *δ* exerts a positive effect on the ${\mathcal R_{0}}$, and *α* has a negative effect on the ${\mathcal R_{0}}$. Firstly, we can encourage people to complete the treatment to be fully cured by reducing the cost [76]. The state can make more medical insurance to relieve the financial burden and help people heal. Secondly, we can educate people to learn more about TB treatment, to follow the doctor’s plan, and to realize that health is more important than everything.

Strategy 6: It shows that *ϕ* is negatively correlated with both ${\mathcal R_{0}}$ and the total infectious, which means increasing the number of vaccinated people per year can protect people from contracting TB, and thus control TB. Even for the state with little burden of TB, BCG can help prevent TB.

In this paper, the data used for fitting is annual data. Due to the extensive time scale, the accuracy of the model will be reduced. In later research, we will choose monthly data. Although our model take into account factors such as slow-fast process [41–43], vaccination [24–26], reinfection [21–23], reactivated [29, 30] and undiagnosed infection [21, 44], these factors are not enough. We did not take into account factors such as interactions with HIV [27, 28], immigration [77, 78] and drug-resistant TB bacilli [69, 79, 80]. In addition to the aforementioned factors, we also did not consider residents’ medical expenditure and awareness of disease control to discuss the prevention and control measures in this study. As for the deficiencies in the study, we will analyze and discuss them in the follow-up study.

## Conclusion

In general, from the analysis, it is evident that, similar to TB studies elsewhere, the prevalence of TB in the United States is heavily influenced by exposure, vaccination, and treatment effectiveness. In our study, we also found that chemoprophylaxis affects the prevalence of TB more than other factors. However, each coin has two sides. Chemoprophylaxis has certain harm to the human body [72], so we should research new and better measures to prevent and control TB. Based on the analysis, we propose some strategies to control and eliminate TB in two ways: prevention and treatment. Chemoprophylaxis stands out as the one that can greatly control TB with some side effects. Accordingly, we should do much research to find better Chemoprophylaxis.

In fact, because the recovered infectious may relapse, the difficulty of diagnosing and treating TB is hard to control. When all the control measures are implemented together (see Fig. 5, solid black line), the basic reproduction number of the model (1) is 0.6915<1. However, the case does not disappear. Instead, the system stabilizes to the endemic equilibrium. The measures we propose may not eliminate TB, but they are critically useful for controlling the epidemic of TB. According to the latest report, in the announcement came at the first WHO Global Ministerial Conference on Ending Tuberculosis in the Sustainable Development Era, 75 ministers agreed to take urgent measures to end TB by 2030 [81]. From our analysis, it is difficult to end TB until 2030 under the existing conditions (Fig. 5). Thus, we should find more and better methods to eradicate TB. Although it will be difficult to eliminate TB in a short period of time, we believe that in the future, with advanced technologies, TB can be eliminated.

## \thelikesection Appendix

It is easy to see that the model (1) always has a disease-free equilibrium *P*_0_, and the disease-free equilibrium, which are the solutions of the algebraic equations:

7 $$\begin{array}{@{}rcl@{}} {\begin{aligned} \left\{ {\begin{array}{*{20}{l}} {\Lambda \chi + \varphi S - (\phi + \mu)V - \varepsilon vV{{ = }}0},\\ {\Lambda (1 - \chi) + \phi V + (1 - {q_{1}} - {q_{2}})\gamma R - \varphi S - vS - \mu S{{ = }}0},\\ {\varepsilon vV + (1 - {p_{1}} - {p_{2}})vS + {q_{2}}\gamma R + \rho J - (1 - r)kE - \mu E{{ = }}0},\\ {{p_{1}}vS + h(1 - r)kE + \theta J + {q_{1}}\gamma R + \alpha L - (\delta + g)I - (\mu + {d_{1}})I{{ = }}0},\\ {(1 - h)k(1 - r)E + {p_{2}}vS - (\theta + \rho)J - (\mu + {d_{2}})J{{ = }}0},\\ {\delta I - (\alpha + \omega)L - (\mu + {d_{3}})L{{ = }}0},\\ {gI + \omega L - (\gamma + \mu)R{{ = }}0},\\ {I = 0}. \end{array}} \right. \end{aligned}} \end{array} $$

The disease-free equilibrium *P*_0_:

8 $$ \begin{aligned} {P_{0}} &= ((\varphi \Lambda + \mu \chi \Lambda)/(\mu \varphi + \mu \phi + {\mu^{2}}),\\&(\mu \Lambda + \phi \Lambda - \mu \chi \Lambda)/(\mu \varphi + \mu \phi + {\mu^{2}}),0,0,0,0,0). \end{aligned}  $$

The next-generation matrix approach is applied to calculate the basic reproduction number ${\mathcal {R}_{0}}$ [49]. For this purpose, we set *X*=(*V*,*S*,*E*,*I*,*J*,*L*,*R*), there are $\dot {X}=F-Q$, where:

9 $$ F = {\left({\begin{array}{*{20}{c}} {0,}&{0,}&{(1 - {p_{1}} - {p_{2}})vS + \varepsilon vV,}&{{p_{1}}vS,}&{{p_{2}}vS,}&{0,}&0 \end{array}} \right)^{T}},  $$

and

10 $$ {\begin{aligned} Q = \left({\begin{array}{*{20}{c}} { - (\Lambda \chi + \varphi S - (\phi + \mu)V - \varepsilon vV)}\\ { - (\Lambda (1 - \chi) + \phi V + (1 - {q_{1}} - {q_{2}})\gamma R - \varphi S - vS - \mu S)}\\ { - ({q_{2}}\gamma R + \rho J - (1 - r)kE - \mu E)}\\ { - (h(1 - r)kE + \theta J + {q_{1}}\gamma R + \alpha L - (\delta + g)I - (\mu + {d_{1}})I)}\\ { - ((1 - h)k(1 - r)E - (\theta + \rho)J - (\mu + {d_{2}})J)}\\ { - (\delta I - (\alpha + \omega)L - (\mu + {d_{3}})L)}\\ { - (gI + \omega L - (\gamma + \mu)R)} \end{array}} \right). \end{aligned}}  $$

Jacobian matrices $\mathcal {F}$ and $\mathcal {Q}$ at the disease-free equilibrium of *F* and *Q* are calculated respectively:

$${\begin{aligned} &\mathcal{F}=\left.\frac{\partial F(V, S, E, I, J, L, R)}{\partial(V, S, E, I, J, L, R)}\right|_{P_{0}}\\ &=\left[\begin{array}{ccccccc} 0 & 0 & 0 & 0 & 0 & 0 & 0 \\ 0 & 0 & 0 & 0 & 0 & 0 & 0 \\ F_{1} & F_{2} & F_{3} & F_{4} & F_{5} & F_{6} & F_{3} \\ W_{1} & p_{1} A_{1}+W_{1} & W_{1} & \frac{S \beta_{1} p_{1}}{N}+W_{1} & \frac{S \beta_{2} p_{1}}{N}+W_{1} & \frac{S \beta_{3} p_{1}}{N}+W_{1} & W_{1} \\ W_{2} & p_{2} A_{1}+W_{2} & W_{2} & \frac{S \beta_{1} p_{2}}{N}+W_{2} & \frac{S \beta_{2} p_{2}}{N}+W_{2} & \frac{S \beta_{3} p_{2}}{N}+W_{2} & W_{2} \\ 0 & 0 & 0 & 0 & 0 & 0 & 0 \\ 0 & 0 & 0 & 0 & 0 & 0 & 0 \end{array}\right] \end{aligned}} $$

where:

$$ \begin{array}{l} F_{1}=\varepsilon A_{1}+S\left({p}_{1}+{p}_{2}-1\right) {A}_{2}-V \varepsilon A_{2} \\ F_{2}=S\left({p}_{1}+{p}_{2}-1\right) {A}_{2}-V \varepsilon A_{2}-\left({p}_{1}+{p}_{2}-1\right) {A}_{1} \\ F_{3}=S\left({p}_{1}+{p}_{2}-1\right) {A}_{2}-V \varepsilon A_{2} \\ F_{4}=S\left({p}_{1}+{p}_{2}-1\right) {A}_{2}-V \varepsilon A_{2}-\frac{S \beta_{1}\left({p}_{1}+{p}_{2}-1\right)+{V} \beta_{1} \varepsilon}{N} \\ F_{5}=S\left({p}_{1}+{p}_{2}-1\right) {A}_{2}-V \varepsilon A_{2}-\frac{S \beta_{2}\left({p}_{1}+{p}_{2}-1\right)+{V} \beta_{2} \varepsilon}{N} \\ F_{6}=S\left({p}_{1}+{p}_{2}-1\right) {A}_{2}-V \varepsilon A_{2}-\frac{S \beta_{3}\left({p}_{1}+{p}_{2}-1\right)+{V} \beta_{3} \varepsilon}{N} \end{array}\notag  $$

and

$$ \begin{array}{l} W_{1}=-S p_{1} A_{2} \\ W_{2}=-S p_{2} A_{2} \\ A_{1}=\frac{I \beta_{1}+J \beta_{2}+L \beta_{3}}{N} \\ A_{2}=\frac{I \beta_{1}+J \beta_{2}+L \beta_{3}}{N^{2}} \\ N=V+S+E+I+L+J+R \end{array}\notag  $$

$${\begin{aligned} \begin{array}{l} \mathcal{Q}=\left.\frac{\partial Q(V, S, E, I, J, L, R)}{\partial(V, S, E, I, J, L, R)}\right|_{P_{0}} \\ \,=\,\left[\!\!\!\!\begin{array}{ccccccc} Q_{1} & -\phi-M & -M & \frac{V \beta_{1} \varepsilon}{N}-M & \frac{V \beta_{2} \varepsilon}{N}-M & \frac{V \beta_{3} \varepsilon}{N}-M & -M \\ -\psi-S A_{2} & Q_{2} & -S A_{2} & \frac{S \beta_{1}}{N}-S A_{2} & \frac{S \beta_{2}}{N}-S A_{2} & \frac{S \beta_{3}}{N}-S A_{2} & Q_{8} \\ 0 & 0 & Q_{3} & 0 & \-\rho & 0 & -\gamma q_{2} \\ 0 & 0 & h k(r-1) & Q_{5} & -\theta & -\alpha & -\gamma q_{1} \\ 0 & 0 & Q_{4} & 0 & Q_{6} & 0 & 0 \\ 0 & 0 & 0 & -\delta & 0 & Q_{7} & 0 \\ 0 & 0 & 0 & -g-\omega & 0 & 0 & \gamma+\mu \end{array}\right] \end{array} \end{aligned}} $$

where

$$ \begin{array}{l} M=V \varepsilon A_{2} \\ Q_{1}=\mu+\psi+\varepsilon A_{1}-M \\ Q_{2}=\mu+\phi+A_{1}-S A_{2} \\ Q_{3}=\mu-k(r-1) \\ Q_{4}=-k(h-1)(r-1) \\ Q_{5}=d_{1}+\delta+g+\mu \\ Q_{6}=d_{2}+\mu+\rho+\theta \\ Q_{7}=\alpha+d_{3}+\mu+\omega \\ Q_{8}=\gamma\left(q_{1}+q_{2}-1\right)-S A_{2} \end{array}\notag  $$

then, one can obtain $G=\mathcal {F}\mathcal {Q}^{-1}$. The basic reproduction number $R_{0}=\max \left \{\lambda _{\mathcal {R}_{0}}\right \}$, where $\lambda _{\mathcal {R}_{0}}$ are the eigenvalues of *G*. Because ${\mathcal {R}_{0}}$ is complex, we don’t give the detailed expression, and compute it via Matlab (the Mathworks, Inc.).

## Data Availability

The data that support the findings of this study are available from the Center for Disease Control and Prevention (CDC) (https://www.cdc.gov/tb/statistics/reports/2017/table1.htm), these network direct data are completely open, and we count these data.

## Abbreviations

TB: Tuberculosis
DESQP: global differential evolution and local sequential quadratic programming
LHS: Latin hypercube sampling
PRCC: partial rank correlation coefficients
ODE: ordinary differential equations
MAPE: the mean absolute percentage error
RMSPE: the root mean square percentage error
AIDS: acquired immunodeficiency syndrome
HIV: human immunodeficiency virus
MTB: Mycobacterium tuberculosis
BCG: Bacillus Calmette - Guerin
China’s CDC: Chinese Center for Disease Control and Prevention
WHO: The World Health Organization
SQP: sequence quadratic program
SA: sensitivity analysis
UA: uncertain sensitivity analysis
TST: tuberculin skin test
Th1: T-helper 1
